# Educating for environmental transition: the summer school on microplastics

**DOI:** 10.1007/s11356-025-37253-y

**Published:** 2025-12-04

**Authors:** Vera I. Slaveykova, Thorbjørn J. Andersen, Tomasz Błasiak, Andrea Cararo, Matea Marelja, Marco Parolini, Nicole R. Posth, David Siaussat, Lynn Sorrentino

**Affiliations:** 1 Faculty of Sciences, University of Geneva, 66, Bvd. Carl-Vogt, 1211 Geneva, Switzerland; 2 Faculty of Science, University of Copenhagen, IGN, Øster Voldgade 10, 1350 Copenhagen, Denmark; 3 Institute for Environmental Sciences, University of Geneva, 66, Bvd Carl-Vogt, 1211 Geneva, Switzerland; 4 United Nations Environment Programme, 8-14, Avenue de La Paix, 1211 Geneva, Switzerland; 5 University of Milan, Via Celoria 26, 20133 Milan, Italy; 6 Institut d’Ecologie et Des Sciences de L’Environnement de Paris (iEES-Paris), Sorbonne Université, 75005 Paris, France; 7 International Union for Conservation of Nature (IUCN), 28 Rue Mauverney, 1196 Gland, Switzerland

**Keywords:** Plastics, Environmental pollution, Human health, Science-policy interface, Education, World Café

## Abstract

Plastics are deeply embedded in modern life, but their degradation releases micro- and nanoplastics (MNPs) into ecosystems. These persistent particles are found everywhere, from oceans to the human body, and raise growing concerns about environmental and human health, biodiversity, and sustainability implications. Despite increasing awareness, effective responses to MNP pollution remain limited by unresolved challenges in scientific monitoring, policymaking, and public engagement. Addressing these challenges, the summer school on "Microplastics: From Environmental Impact to Policy, Innovation, and Public Awareness" held in June 2025 in Geneva, Switzerland, exemplifies an innovative educational model. The program was multi- and interdisciplinary, action-oriented, internationally collaborative, and rooted in local contexts. Focusing on microplastic pollution in aquatic environments, it brought together participants from 15 countries to explore the nexus of science, policy, governance, innovation, and public engagement. This contribution reflects the summer school’s design and outcomes, highlighting its promise as a model for advancing next-generation environmental education as well as discussing some of the key challenges.

## Teaching on microplastics in the “age of plastics”

In an age defined by both plastics materials abundance (Rangel-Buitrago et al. 2022; Stubbins et al. 2021) and environmental fragility, microplastics have emerged as a symbol of the unintended consequences of technological progress. According to the Organization for Economic Cooperation and Development (OECD), global plastics use is projected to triple between 2019 and 2060, rising from 460 to 1 321 Mt (OECD (2022)). Concomitantly, plastic waste release is also increasing, with a significant portion entering the environment due to mismanagement, inadequate recycling infrastructure, and consumption patterns (Kibria et al. 2023; Singh and Walker 2024). According to United Nation Environmental Programme (UNEP), every year 19–23 million tons of plastic waste leaks into aquatic ecosystems, polluting lakes, rivers, and seas (Borrelle et al. 2020; UNEP 2025). As plastic waste breaks down into minuscule fragments, it gives rise to a novel class of pollutants—micro- and nanoplastics (MNPs) (Kumar et al. 2023). These particles, measuring under 5 mm and often much smaller (Hartmann et al. 2019), have become virtually ubiquitous. In addition to their small size and high reactivity, MNPs are chemically complex and diverse containing more than 16,000 known plastic chemicals, including 4200 chemicals of concern (Monclús et al. 2025). They have been found across the planet: from the deepest ocean trenches to mountain peaks, from agricultural soils to atmospheric currents, and even within human organs and tissue (Nihart et al. 2025; Thompson et al. 2024; Zhao et al. 2025). The growing presence of MNPs in both environmental and biological systems has raised concerns about their potential impacts on the ecosystems and human health (Bao et al. 2024; Koelmans et al. 2022; Thompson et al. 2024). Ecologically, they may alter food web dynamics, affect species’ reproductive health, and transport toxic chemicals across ecosystems (Basumatary et al. 2025; Chanda et al. 2024; MacLeod et al. 2021). From a human health perspective, the possible implications of long-term exposure remain insufficiently understood, but studies increasingly suggest potential links to inflammation, endocrine disruption, and cellular damage (Alijagic et al. 2024). Despite rising concerns, significant challenges remain in detecting, quantifying, and studying the effects of MNPs. These challenges include the lack of standardized methodologies, limitations in analytical techniques to detect particles at the nanoscale, and difficulty in distinguishing plastic particles from natural particulates (Ivleva 2021). Moreover, variability in polymer types, shapes, and chemical additives further complicates environmental fate and (eco)toxicological assessments, as well as risk evaluations (Monclús et al. 2025). As such, multi- and interdisciplinary efforts are urgently needed to advance detection technologies, assess risks, and inform regulatory frameworks to address these environmental and health concerns. Beyond these scientific and policy challenges, MNPs also pose an educational challenge. How can we teach students to engage in issues that are complex, interdisciplinary, and deeply connected to everyday life? Are young people, students, and the broader public adequately informed about these pressing concerns? Education is critical in this context, as raising awareness can influence behavior, encourage reduced plastic use, improve waste management, and promote engagement in community or policy initiatives. Linking knowledge through education supports the societal changes necessary to complement technological and regulatory solutions to MNPs pollution.

The summer school on microplastics at the University of Geneva was launched in June 2025 to tackle the complex challenges posed by plastic pollution. This initiative was supported by the SEED call of the 4EU + Alliance under the auspices of flagship 4: Environmental transitions. The project titled “Microplastics: From Environmental Impact to Policy, Innovation, and Public Awareness (MIRACLE)” (Slaveykova et al. 2024) was led by the University of Geneva, Switzerland, in collaboration with the University of Copenhagen (Denmark), the University of Milano (Italy); and Sorbonne University (France). The program brought together 40 participants from 15 countries in Europe and beyond to examine MNPs pollution in aquatic environments through a unique multi-, inter-, and trans- disciplinary approach. The learning objectives of the program were threefold: (i) to boost student engagement and learning on this crucial and high current interest topic, (ii) to offer students the chance to develop problem-solving skills by tackling real global issues in collaboration with professionals, and (iii) to facilitate effective teamwork among students from diverse backgrounds, while applying their knowledge in environmental management and communication. The program was designed to develop critical competencies based on an integrated, systems-oriented approach, an approach that educates the next generation to think across disciplinary boundaries, act collaboratively, and design resilient environmental strategies.

## Multi- and inter-disciplinary by design

There is a consensus that to address the global issue of MNPs pollution characterized by high complexity and multidimensionality, an innovative, inter- and transdisciplinary, and multisectoral approaches must be adopted (Kammerer et al. 2025; Lang et al. 2012). Transdisciplinary approaches are essential for tackling complex sustainability challenges by fostering collaboration between science and society in the joint production of knowledge (Lang et al. 2012). As an alternative to traditional, disciplinary approaches, this curriculum embraced an inter- and multi-disciplinary framework that encouraged students to think across boundaries, drawing from the natural sciences, life sciences, social and policy studies, as well as material and product design. Rather than focusing solely on disciplinary achievement or lecture-based instruction, the program integrated dialogue-based and collaborative learning through a team-based activities. Through such integrative and participatory designs, students develop systems thinking, critical reflection, and a sense of how to link scientific understanding with everyday action towards sustainable future.

The summer school on microplastics was a 1-week intensive in-presence program designed for bachelor’s, master’s, and PhD students, as well as early-career professionals, typically aged 23–35. Recruitment was global, targeting students and early-career professionals interested in environmental issues, with scholarships provided for international participants from 4EU + Alliance. The student cohort reflected a diverse academic background, encompassing bachelor’s degrees in physics, chemistry, political science, and international relations; master’s studies in environmental sciences, law and sustainable development, and environmental change and global sustainability; and doctoral research in environmental sciences. This diversity fostered an interdisciplinary learning environment that encouraged integrative thinking and collaboration across fields. Students were primarily motivated by the program’s multi-, inert-, and transdisciplinary orientation and its emphasis on linking scientific knowledge with societal challenges and sustainable development goals. The focus on MNPs, a topic of growing global relevance and urgency, further enhanced the program’s appeal, offering an opportunity to engage with a complex environmental issue situated at the intersection of science, policy, and everyday life.

**Fig. 1:**
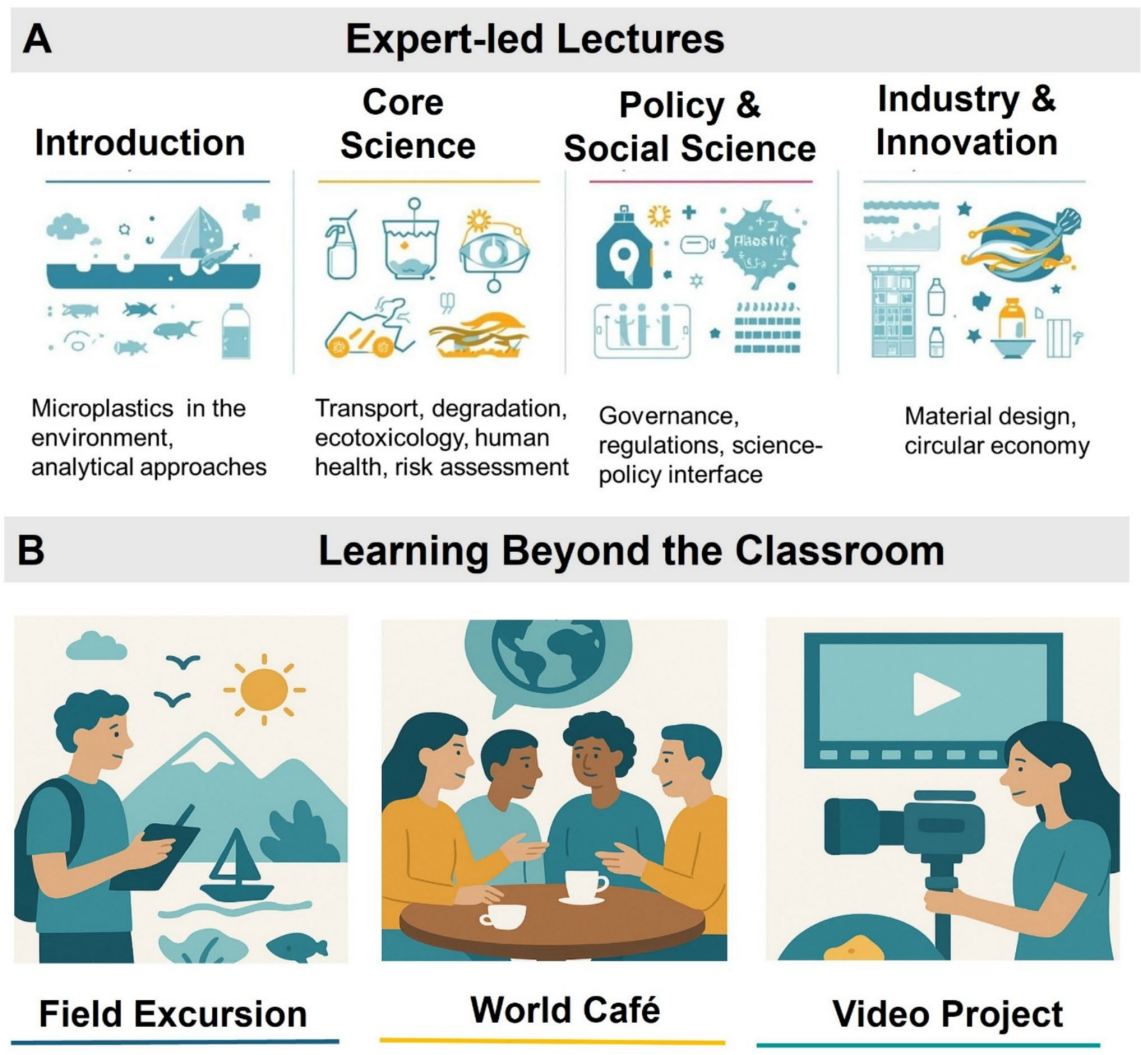
Overview of the summer school program structure highlighting expert-led lecture modules and learning beyond the classroom activities

The school’s program was structured around a combination of expert-led lectures, interactive workshops, and hands-on field activities, delivered by a diverse faculty including university professors, policy experts, and representatives from international organizations. The curriculum was organized into sequential modules (Fig. 1), beginning with foundational knowledge on global plastic production, the relevance of microplastic pollution, and existing techniques and challenges for their detection. Students received core instruction on plastic degradation processes, environmental transport mechanisms, and ecotoxicological impacts on aquatic ecosystems, as well as the potential implications for human health, linking environmental exposure to physiological and toxicological effects.

Expanding beyond the natural sciences, the program integrated social sciences and policy studies, examining global and regional governance, regulatory frameworks, and the science-policy interface. An industry-focused perspective encouraged students to explore innovative solutions, including biodegradable materials and circular economy approach to reduce plastic waste. To reinforce learning and foster collaborative problem-solving, these modules were complemented by field-, and dialogue-based activities, such as World Café discussion, hands-on environmental survey, and a creative movie-making group project, allowing students to apply knowledge in practical contexts.

## Learning beyond the classroom

Building on the program’s expert-led lecture modules, students engaged in experiential activities—field excursion, World Café session, and video-making projects—that allowed them to apply knowledge, collaborate, and communicate insights in real-world contexts.

1. Field excursion: The field excursion offered students practical, immersive experience in environmental monitoring, focusing on the presence and impact of plastic pollution along the urban shoreline of Lake Geneva. Conducted in a highly managed freshwater ecosystem, this activity provided participants with the opportunity to engage directly in on-site sampling of both microplastic and macroplastic debris. Students were introduced to field-based research methodologies, including current sampling protocols, techniques for identifying and quantifying plastic waste, and methods for extracting microplastics from environmental matrices, such as sand and sediment. Beyond technical skill development, the excursion served as a platform for critical reflection on the complexity of pollution in urban lacustrine environments. Through hands-on engagement, students gained a deeper understanding of the environmental consequences of plastic pollution and the challenges of detecting, analyzing, and mitigating such pollutants in real-world contexts. The activity also emphasized the importance of long-term environmental monitoring, inter-site data comparison, and accurate scientific reporting, skills essential for contributing to ongoing research and policy development related to plastic pollution. By combining experiential learning with scientific inquiry, the field trip reinforced key themes of the course, including environmental systems thinking, evidence-based observation, and interdisciplinary problem-solving.
2. World Café: The World Café is a method offering a platform for dynamic and facilitated dialogue that brought together experts from academia, government agencies, and non-governmental organizations to engage students in sector-specific discussions on microplastic pollution. Covering diverse sources of microplastics, including tire abrasion, plastic packaging, synthetic textiles, personal care products, cosmetics, and bioplastics, the topics enabled students to explore the multifaceted nature of plastic-related environmental challenges. The informal, yet structured format promoted open, collaborative inquiry, fostering meaningful exchanges between students and professionals with real-world expertise. By rotating through expert-led discussion tables, students gained insights into sectoral priorities, regulatory constraints, technological innovations, and policy considerations. This engagement not only deepened student understanding of microplastics from multiple perspectives. It also bridged the gap between academic learning and professional practice, reinforcing the value of interdisciplinary dialogue and systems thinking in addressing complex environmental issues.
3. Video project: Students worked in teams to produce 1-min public education videos on microplastic topics. This project was designed to cultivate collaborative learning, encourage creativity, and enhance science communication skills. Participants worked in small groups of four students to conceptualize, script, and produce a 1-min video that connects key scientific and societal aspects of MNPs pollution in aquatic environments. The exercise encouraged students to present complex information into engaging stories using visual media, while also reinforcing systems thinking by illustrating the interrelated nature of environmental problems. The videos showcased students’ capacity to reach broad audiences with clear, impactful narratives—underscoring the vital role of communication, innovation, and critical analysis in tackling pressing ecological issues.

The summer school moved beyond the traditional educational approach and used a combination of several methods and approaches geared toward active engagement of students, who can thus learn more effectively by putting their knowledge into practice. This integrated approach fostered dynamic learning loops—linking scientific theory with practical application, critical analysis with public communication, and individual reflection with group collaboration. One participant noted that the “*World* *Café* *was* *very* *interesting,* *and the video project was a fun way to deepen our knowledge* *and engage with the topics with a hands-on approach.*” By emphasizing active participation and cross-sectoral perspectives, the program cultivated the skills, mindsets, and cooperative attitudes essential for addressing the complex and interconnected environmental issues of the twenty-first century.

## Role of partnership

Set in Geneva, the program takes advantage of the city’s strategic position as a global hub for environmental governance. With guest speakers from institutions, such as the United Nations Environment Program, the International Union for Conservation of Nature, the World Economic Forum, and Switzerland’s Federal Office for the Environment, participants were exposed to the real-world interfaces between science, policy, and diplomacy. Geneva’s unique institutional density allowed students to engage directly with actors involved in shaping global environmental agendas, providing an invaluable lens into how scientific evidence is translated and operationalized within international decision-making processes. The experience provided an important lesson for future researchers and practitioners: that addressing complex environmental challenges requires not only robust science, but also the capacity to navigate political, institutional, and cultural boundaries. The experience of studying in Geneva, “a global hub for international organizations”, was also described as deeply impactful. As one student shared, “*Studying in Geneva, home to many international organizations, was a meaningful experience. It provided valuable opportunities for networking and gaining new insights. Hearing directly from leading academics and experts from international organizations was particularly enriching*.”

The summer school offered more than just an opportunity to study microplastics; it challenged participants to rethink leadership amid escalating planetary crises. In a world shaped by climate change, biodiversity loss, and chemical pollution, the value of environmental education is increasingly measured by its ability to develop transformative skills, such as systems thinking, critical reflection, effective action, and clear communication, rather than the mere quantity of information conveyed.

This summer school was part of the 4EU + Alliance project, MIRACLE. Through initiatives like this, the 4EU + Alliance has established a dynamic educational ecosystem that crosses national, disciplinary, and institutional borders, enabling meaningful collaboration between students and educators across Europe. This holistic approach advances the goals of the European higher education area by fostering joint academic initiatives, shared learning opportunities, and increased mobility. The summer school exemplified this vision, showcasing the power of transnational cooperation to enhance intercultural competence, interdisciplinary thinking, and academic collaboration. The success of the summer school highlights the vital role of international academic partnerships in building a more integrated, adaptable, and globally connected European higher education community.

As universities adopt their curricula to address the challenges of our changing environment, this summer school offers a valuable example. Its educational framework promotes interdisciplinary integration, active stakeholder involvement, creative engagement, and collaboration across institutions and borders, with exchange and mobility embedded as essential elements of environmental literacy. These elements align closely with global policy frameworks, such as UNESCO’s Education for Sustainable Development, the European Green Deal’s education priorities, and the UN Decade of Ocean Science. It contributed to education in relation to multiple sustainable development goals (SDG), namely SDG 3—good health and well-being, SDG 4—quality education, SDG 6—clean water, SDG 12—responsible consumption, SDG 14—life under water, and SDG 17—strengthening the partnership for the goals. Ultimately, the summer school exemplified what environmental higher education must increasingly become: not only a transmitter of knowledge, but a catalyst for societal transformation. As this course was offered as a highly intensive summer school program, its structure was intentionally designed to cover a broad range of foundational topics within a limited timeframe. Consequently, a potential area for future development could include the incorporation of more practical components focused on upstream and downstream pollution prevention, circular economy approaches, and transboundary cooperation to enhance applied learning and regional relevance. While the summer school highlighted the potential of multi-, inter-, and transdisciplinary education to address complex sustainability challenges, several lessons emerged. Balancing disciplinary depth with integrative breadth remained a key challenge, as did linking scientific insights more effectively with policy and practice. It is hoped that students will apply their newly acquired knowledge and communication techniques to advance evidence-based decisionmaking, foster public awareness, and drive innovation within their respective professional and academic contexts.

## Conclusion

Microplastic pollution will shape environmental narratives and regulatory landscapes well into the twenty-first century. If we aim at responding effectively, we have to reimagine environmental education itself, not as a passive transmission of facts, but as a living ecosystem of collaboration, action, and learning. The summer school on microplastics stands as a proof-of concept: a place-based, globally connected, multidimensional model for teaching and learning in the “age of plastics.” As MNPs pollution continues to challenge science, policy, and society, we argue that educational innovation is itself a critical policy tool. Preparing future innovators, leaders, and problem-solvers to navigate the uncertainties and urgencies of a rapidly changing planet is not just training for the future of education, it is training for the future of the planet and human society itself. We hope this initiative will inspire similar programs across disciplines and continents because the future of our ecosystems depends not only on innovation, but on education that can keep up with the complexity of the world we have made.

## Data Availability

This is not applicable.

## References

[CR1] Alijagic A, Suljević D, Fočak M, Sulejmanović J, Šehović E, Särndahl E, Engwall M (2024) The triple exposure nexus of microplastic particles, plastic-associated chemicals, and environmental pollutants from a human health perspective. Environ Int 188:108736. 10.1016/j.envint.2024.108736 38759545 10.1016/j.envint.2024.108736

[CR2] Bao L-J, Mai L, Liu L-Y, Sun X-F, Zeng EY (2024) Microplastics on the planet: current knowledge and challenges. Environ Sci Technol Lett 11(12):1262–1271. 10.1021/acs.estlett.4c00603

[CR3] Basumatary T, Biswas D, Boro S, Nava AR, Narayan M, Sarma H (2025) Dynamics and impacts of microplastics (MPs) and nanoplastics (NPs) on ecosystems and biogeochemical processes: the need for robust regulatory frameworks. ACS Omega 10(17):17051–17069. 10.1021/acsomega.5c01175 40352536 10.1021/acsomega.5c01175 PMC12060063

[CR4] Borrelle SB, Ringma J, Law KL, Monnahan CC, Lebreton L, McGivern A, Murphy E, Jambeck J, Leonard GH, Hilleary MA, Eriksen M, Possingham HP, De Frond H, Gerber LR, Polidoro B, Tahir A, Bernard M, Mallos N, Barnes M, Rochman CM (2020) Predicted growth in plastic waste exceeds efforts to mitigate plastic pollution. Science 369(6510):1515–1518. 10.1126/science.aba3656 32943526 10.1126/science.aba3656

[CR5] Chanda M, Bathi JR, Khan E, Katyal D, Danquah M (2024) Microplastics in ecosystems: critical review of occurrence, distribution, toxicity, fate, transport, and advances in experimental and computational studies in surface and subsurface water. J Environ Manage 370:122492. 10.1016/j.jenvman.2024.122492 39307085 10.1016/j.jenvman.2024.122492

[CR6] Hartmann NB, Hüffer T, Thompson RC, Hassellöv M, Verschoor A, Daugaard AE, Rist S, Karlsson T, Brennholt N, Cole M, Herrling MP, Hess MC, Ivleva NP, Lusher AL, Wagner M (2019) Are we speaking the same language? Recommendations for a definition and categorization framework for plastic debris. Environ Sci Technol 53(3):1039–1047. 10.1021/acs.est.8b05297 30608663 10.1021/acs.est.8b05297

[CR7] Ivleva NP (2021) Chemical analysis of microplastics and nanoplastics: challenges, advanced methods, and perspectives. Chem Rev 121(19):11886–11936. 10.1021/acs.chemrev.1c00178 34436873 10.1021/acs.chemrev.1c00178

[CR8] Kammerer M, Beer T, Wiedemann R (2025) Taming plastic pollution—a systematic mapping of the literature on plastic policies between 2009 and 2024. Reg Environ Change 25(3):90. 10.1007/s10113-025-02420-x

[CR9] Kibria MG, Masuk NI, Safayet R, Nguyen HQ, Mourshed M (2023) Plastic waste: challenges and opportunities to mitigate pollution and effective management. Int J Environ Res 17(1):20. 10.1007/s41742-023-00507-z 36711426 10.1007/s41742-023-00507-z PMC9857911

[CR10] Koelmans AA, Redondo-Hasselerharm PE, Nor NHM, de Ruijter VN, Mintenig SM, Kooi M (2022) Risk assessment of microplastic particles. Nat Rev Mater 7(2):138–152. 10.1038/s41578-021-00411-y

[CR11] Kumar V, Singh E, Singh S, Pandey A, Bhargava PC (2023) Micro- and nano-plastics (MNPs) as emerging pollutant in ground water: environmental impact, potential risks, limitations and way forward towards sustainable management. Chem Eng J 459:141568. 10.1016/j.cej.2023.141568

[CR12] Lang DJ, Wiek A, Bergmann M, Stauffacher M, Martens P, Moll P, Swilling M, Thomas CJ (2012) Transdisciplinary research in sustainability science: practice, principles, and challenges. Sustain Sci 7(1):25–43. 10.1007/s11625-011-0149-x

[CR13] MacLeod M, Arp HPH, Tekman MB, Jahnke A (2021) The global threat from plastic pollution. Science 373(6550):61–65. 10.1126/science.abg5433 34210878 10.1126/science.abg5433

[CR14] Monclús L, Arp HPH, Groh KJ, Faltynkova A, Løseth ME, Muncke J, Wang Z, Wolf R, Zimmermann L, Wagner M (2025) Mapping the chemical complexity of plastics. Nature 643(8071):349–355. 10.1038/s41586-025-09184-8 40634741 10.1038/s41586-025-09184-8 PMC12240811

[CR15] Nihart AJ, Garcia MA, El Hayek E, Liu R, Olewine M, Kingston JD, Castillo EF, Gullapalli RR, Howard T, Bleske B, Scott J, Gonzalez-Estrella J, Gross JM, Spilde M, Adolphi NL, Gallego DF, Jarrell HS, Dvorscak G, Zuluaga-Ruiz ME, West AB, Campen MJ (2025) Bioaccumulation of microplastics in decedent human brains. Nat Med 31(4):1114–1119. 10.1038/s41591-024-03453-1 39901044 10.1038/s41591-024-03453-1 PMC12003191

[CR16] OECD (2022) G.P.O.P.S.t. OECD Publishing, Paris. 10.1787/aa1edf33-en

[CR17] Rangel-Buitrago N, Neal W, Williams A (2022) The plasticene: time and rocks. Mar Pollut Bull 185:114358. 10.1016/j.marpolbul.2022.114358 36401945 10.1016/j.marpolbul.2022.114358

[CR18] Singh N, Walker TR (2024) Plastic recycling: a panacea or environmental pollution problem. NPJ Mater Sustain 2(1):17. 10.1038/s44296-024-00024-w 39114578 10.1038/s44296-024-00024-w PMC11304528

[CR19] Slaveykova VI, Andersen TJ, Posth NRE, Parolini M, Siaussat D, 2024. MIRACLE : microplastics, from environmental impact to policy, innovation, and public awareness. https://4euplus.eu/4EU-1121.html.

[CR20] Stubbins A, Law KL, Muñoz SE, Bianchi TS, Zhu L (2021) Plastics in the Earth system. Science 373(6550):51–55. 10.1126/science.abb0354 34210876 10.1126/science.abb0354

[CR21] Thompson RC, Courtene-Jones W, Boucher J, Pahl S, Raubenheimer K, Koelmans AA (2024) Twenty years of microplastic pollution research—what have we learned? Science 386(6720):eadl2746. 10.1126/science.adl2746 39298564 10.1126/science.adl2746

[CR22] UNEP, 2025. https://www.unep.org/plastic-pollution.

[CR23] Zhao S, Kvale KF, Zhu L, Zettler ER, Egger M, Mincer TJ, Amaral-Zettler LA, Lebreton L, Niemann H, Nakajima R, Thiel M, Bos RP, Galgani L, Stubbins A (2025) The distribution of subsurface microplastics in the ocean. Nature 641(8061):51–61. 10.1038/s41586-025-08818-1 40307520 10.1038/s41586-025-08818-1 PMC12043517

